# Association of smoking with cartilage loss of knee osteoarthritis: data from two longitudinal cohorts

**DOI:** 10.1186/s12891-023-06953-2

**Published:** 2023-10-13

**Authors:** Ziyuan Shen, Yining Wang, Xing Xing, Graeme Jones, Guoqi Cai

**Affiliations:** 1 Department of Epidemiology and Biostatistics, School of Public Health, Anhui Medical University, Hefei, 230032 Anhui China; 2 Menzies Institute for Medical Research, University of Tasmania, Hobart, TAS 7000 Australia

**Keywords:** Cartilage volume, Inverse probability of treatment weighting, Osteoarthritis, Smoking

## Abstract

**Background:**

Previous studies have been inconsistent concerning the association between smoking and risk of osteoarthritis (OA). This study aimed to explore the associations of smoking status and change in cartilage volume of OA in two longitudinal cohorts.

**Methods:**

Subjects from the Osteoarthritis Initiative cohort (OAI, n = 593) and the Tasmanian Older Adult Cohort (TASOAC, n = 394) were included in this study. For both cohorts, participants were classified into three groups based on their smoking status, namely ‘never’, ‘former’, and ‘current’ smokers. The outcome measures were the annual rate of change of tibiofemoral cartilage volume over 2 years in OAI and of tibial cartilage volume over 2.6 years in TASOAC. Potential confounders were balanced using the inverse probability of treatment weighting (IPTW) method.

**Results:**

Overall, 42.3% and 37.4% of participants were former smokers, and 5.7% and 9.3% were current smokers in the OAI and TASOAC cohorts, respectively. Compared to never smokers, neither former nor current smoking was associated with risk of the annual rate of change of tibiofemoral cartilage volume in OAI (former smoker: β=-0.068%/year, 95% confidence interval [CI] -0.824 to 0.688, p = 0.860; current smoker: β=-0.222%/year, 95% CI -0.565 to 0.120, p = 0.204) and tibial cartilage volume in TASOAC (former smoker: β = 0.001%/year, 95% CI -0.986 to 0.989, p = 0.998; current smoker: β=-0.839%/year, 95% CI -2.520 to 0.844, p = 0.329).

**Conclusions:**

Our findings from two independent cohorts consistently showed that smoking was not associated with knee cartilage loss in older adults.

**Supplementary Information:**

The online version contains supplementary material available at 10.1186/s12891-023-06953-2.

## Introduction

Osteoarthritis (OA) is the most common joint disease that can lead to pain, functional disability, and impaired quality of life [1]. Worldwide, over 300 million individuals have symptomatic OA, and the prevalence is increasing with age [2]. Numerous studies have demonstrated that person-level risk factors (e.g., age, gender, overweight, occupation, and genetics) and joint-level adverse factors (e.g., injury and malalignment) play a role in the development of OA [3–6]. At the population level, understanding lifestyle factors that associated with the development of OA is critically important for enhancing the management of OA.

Smoking causes more than 8 million deaths each year and is a major risk factor for lung cancer as well as cardiovascular and respiratory diseases [7, 8]. However, the association between smoking and OA is ambiguous. While a recent Mendelian randomization study showed that smoking may increase OA risk [9], many population-based studies observed a protective effect of smoking on OA [10, 11]. In a meta-analysis of observational studies, an inverse association between smoking and OA was found only in hospital-based case-control studies but not in cohort or cross-sectional studies [12]. In a more recent meta-analysis, however, the authors showed a consistent inverse association between smoking and OA, irrespective of study setting and design [13]. It has been indicated that the association between smoking and OA may be biased by methodologic issues and residual confounding, especially obesity [14]. Moreover, most studies have focused on evaluating the effect of smoking on the radiographic progression of OA [13], which is less sensitive than structural abnormalities measured by magnetic resonance imaging (MRI) [15]. One small study used a semiquantitative measure of cartilage score on MRI showing that current smokers (n = 19) had greater cartilage loss and more severe knee pain than non-current smokers in men with symptomatic knee OA [16], and another study had similar findings that smoking led to greater cartilage loss and defect in a younger population with a family history of OA [17].

In this study, we aimed to evaluate the effect of smoking on cartilage loss of knee OA in two longitudinal cohorts.

## Methods

### Study design and participants

The report of this study followed the Strengthening the Reporting of Observational Studies in Epidemiology (STROBE) statement [18]. We used data from the Osteoarthritis Initiative (OAI) and the Tasmanian Older Adult Cohort (TASOAC).

The OAI is a publicly available multicenter longitudinal project conducted in four centers in the United States (https://nda.nih.gov/oai/). A total of 4,796 participants (age 45 to 79 years) with or at increased risk of symptomatic knee OA were enrolled. For this study, we included 593 participants who had data on smoking status and underwent MRI assessments at baseline and the 2-year follow-up. Ethics approvals were obtained from the institutional review board at each of the four clinical centers that recruited OAI participants. All participants provided informed consent.

The Tasmanian Older Adult Cohort (TASOAC) is a prospective study in southern Tasmania, Australia [19]. A total of 1099 participants aged 50 to 79 were selected using a sex-stratified random sampling in southern Tasmania (population 229,000), 98% of whom were white. The current study included 394 participants who had data on smoking status and underwent MRI assessments at baseline and the 2.6-year follow-up. All participants provided written informed consent, and the Southern Tasmanian Health and Medical Human Research Ethics Committee approved this study.

### Outcome measures

In the OAI cohort, data on cartilage volume at baseline and 2 years follow-up were obtained from the Osteoarthritis Biomarkers Consortium FNIH Project, which were computed using a fully automatic computer-based framework called KneeIQ [20]. Three measurements of cartilage volume were conducted for the medial femur and medial tibial plateau, lateral femur and lateral tibial plateau, and the patellar site. Cartilage volume was analyzed in the entire knee and subregions including the medial and the lateral tibiofemoral compartments and the medial and the lateral condyles and plateaus. In this study, we summed the quantifications of the cartilage volume of each participant, and the annual rate of change over 2 years (%/year) was measured as 100 × [(baseline cartilage volume − follow-up cartilage volume)/baseline cartilage volume]/time between two scans in years.

In the TASOAC cohort, MRI of the right knee was performed for each participant at baseline and 2.6 years follow-up. Tibial (medial and lateral) cartilage volume was assessed paired with known chronology using T1-weighted fat-suppressed three-dimensional gradient recall acquisition in the steady state, flip angle 55°, repetition time 58 msec, echo time 12 msec, field of view 16 cm, 60 partitions and 512 × 512 matrix. The intra-observer coefficient of variation (CV) for cartilage volume measures was 2.1% for the medial tibial and 2.2% for the lateral tibial [21]. Annual rate of change in total tibial cartilage volume over 2.6 years (%/year) was measured as 100 × [(baseline cartilage volume − follow-up cartilage volume)/baseline cartilage volume]/time between two scans in years.

#### Smoking status

Smoking history was recorded at baseline by self-reported questionnaire in both cohorts. In the OAI, smoking status was defined as never, former, and current smoker, based on two questions: ‘Have you smoked at least 100 cigarettes (five packs) in your entire life?’, and ‘Do you smoke cigarettes now?’. In the TASOAC, the same smoking status (i.e. never, former, and current smoker) was defined based on two questions: ‘Have you ever been a ‘regular smoker’ (i.e. someone who has smoked at least 7 cigarettes, cigars or pipes every week for at least 3 months)?’, and ‘Are you currently a ‘regular smoker’?’.

#### Covariates

Covariates were selected based on previous studies for their potential confounding effects on the association between smoking and OA [22–24]. Age (year), gender, body mass index (BMI, kg/m^2^), radiographic OA (based on the Kellgren-Lawrence grades) [25], education level, income, marital status, race (white, black, and other), physical activity (the Physical Activity Scale for the Elderly) [26], history of knee surgery (other than knee replacement), history of knee injury, and family history of OA were selected as covariates in the OAI cohort.

The same covariates were used in the TASOAC cohort, with the following exceptions: (1) most participants were white and thus race was not selected as a covariate; (2) marital status, income, and family history of OA were not recorded; (3) history of knee injury was evaluated at the 2.6-year follow-up rather than at baseline; (4) physical activity was evaluated as steps/day using a pedometer (Omron HJ–003 and HJ-102, Omron Healthcare, Kyoto, Japan). Participants were instructed to wear a pedometer for 7 consecutive days with a 6-month interval, and the average steps were calculated at both time points to account for seasonal variation [27]; (5) radiographic OA was defined as the presence of any joint space narrowing (JSN) or osteophytes, according to the Osteoarthritis Research Society International (OARSI) atlas [28].

### Statistical analysis

Baseline characteristics were presented as mean (standard deviation, SD) and n (%). Analysis of variance, Chi-square test and Kruskal-Wallis test were utilized for the comparison of different groups. Inverse probability of treatment weighting (IPTW) method based on propensity scores (PS) was used to achieve covariate balance between the three groups at baseline for each of the two cohorts [29, 30]. PS was the probability of being in one of the three groups conditional on observed covariates and was calculated using multinomial logistic regression models, with smoking status as a dependent variable. In the OAI cohort, twelve variables used for calculating PS, which included age, gender, BMI, radiographic OA, education level, income, marital status, race, physical activity, history of knee surgery, history of knee injury, and family history of OA. In the TASOAC cohort, eight variables were used for calculating PS, which included age, gender, BMI, education level, physical activity, radiographic OA, history of knee surgery, and history of knee injury. The weights were obtained by taking the inverse of the probability of being in one of the three smoking groups. The truncation value was set at 0.01 to minimize the impact of extreme weights, where the weights at the 1st to 99th percentiles were kept [31]. Moreover, the stabilized weights approach was used to increase statistical efficiency and attain better coverage of confidence intervals [32]. The standardized mean difference (SMD) was measured to determine the covariate balance pre- and post-IPTW, with SMD > 0.1 indicating an imbalance. Linear regression models were used to assess the associations of smoking status with the annual percentage change of cartilage volume for the OAI and TASOAC cohorts, respectively. Models adjusted for PS-based weights and the imbalanced covariates after weighting (i.e. SMD > 0.1). Considering the potential moderating effect of age and family history of OA [17], the interactions of smoking status with age in groups (< 60; 60–70; ≥70) and family history of OA were evaluated, in which the interaction between smoking status and family history of OA was only evaluated in the OAI due to data availability. The assumptions of linear regression were tested for each of these models.

Two sensitivity analyses were conducted in this study. First, it has been suggested that BMI may be a mediator for the association between smoking and OA and that adjusting for BMI may introduce collider-stratification bias [14]. Therefore, we performed IPTW by not balancing baseline BMI. Second, multiple imputation with chained equations (MICE) was adopted to account for missing data (0.17–5.23% missing in OAI, and 0.25–4.82% missing in TASOAC), assuming missing at random. Five imputed datasets were created and the IPTW approach was adopted for each imputed dataset, and the final results were pooled using Rubin’s rules.

All statistical analyses were performed by R software (version 4.2.0; http://www.Rproject.org). Statistical significance was set at a *p* value of ≤ 0.05 (two-tailed).

## Results

### Participants

In the OAI cohort, 593 participants (59.3% female, mean age 61.6 ± 8.9 years [range 45.0–79.0]) were enrolled in this study, and the baseline characteristics splitting by smoking status were shown in Additional file 1. Table 1 summarized the baseline characteristics of 546 participants with complete data. The main between-group difference was age, sex, BMI, income, marital status, race, educational level, and history of knee surgery.

**Table 1:** Baseline Characteristics of OAI cohort, Pre- and Post-IPTW

	*Pre-IPTW*					*Post-IPTW*			
	Never (n = 284)	Former (n = 231)	Current (n = 31)	*SMD*		Never	Former	Current	*SMD*
Age (mean (SD))	61.28 (8.98)	62.84 (8.48)	56.97 (8.21)	0.461		61.79 (9.13)	61.76 (8.64)	64.26 (10.88)	0.168
Sex									
Male	108 (38.0)	110 (47.6)	10 (32.3)	0.211		119.0 (41.9)	95.1 (41.2)	6.4 (22.3)	0.286
Female	176 (62.0)	121 (52.4)	21 (67.7)			165.0 (58.1)	135.9 (58.8)	22.5 (77.7)	
BMI (mean (SD))	30.66 (4.92)	30.81 (4.49)	31.84 (4.73)	0.166		30.74 (4.94)	30.76 (4.61)	28.88 (5.12)	0.254
Income (%)									
<10k	9 (3.2)	4 (1.7)	3 (9.7)	0.478		9.0 (3.2)	8.0 (3.5)	0.6 (2.2)	0.204
10-25k	36 (12.7)	25 (10.8)	7 (22.6)			34.5 (12.1)	28.3 (12.3)	4.7 (16.1)	
25-50k	71 (25.0)	71 (30.7)	10 (32.3)			78.0 (27.5)	64.4 (27.9)	7.2 (24.8)	
50-100k	98 (34.5)	82 (35.5)	9 (29.0)			101.2 (35.6)	81.4 (35.2)	12.8 (44.4)	
>100k	70 (24.6)	49 (21.2)	2 (6.5)			61.4 (21.6)	49.0 (21.2)	3.6 (12.5)	
Marital status (%)									
Married or partnered	193 (68.0)	156 (67.5)	12 (38.7)	0.509		188.2 (66.3)	152.7 (66.1)	18.9 (65.3)	0.081
Widowed	18 (6.3)	14 (6.1)	2 (6.5)			18.4 (6.5)	14.7 (6.4)	2.0 (6.9)	
Divorced	42 (14.8)	46 (19.9)	9 (29.0)			49.9 (17.6)	40.6 (17.6)	5.8 (20.0)	
Separated	5 (1.8)	2 (0.9)	1 (3.2)			4.1 (1.4)	3.3 (1.4)	0.2 (0.6)	
Never married	26 (9.2)	13 (5.6)	7 (22.6)			23.3 (8.2)	19.7 (8.5)	2.1 (7.2)	
Education level (%)									
Less than high school	11 (3.9)	5 (2.2)	1 (3.2)	0.432		9.3 (3.3)	7.2 (3.1)	0.3 (1.0)	0.353
High school	42 (14.8)	29 (12.6)	4 (12.9)			39.7 (14.0)	33.4 (14.5)	3.9 (13.4)	
Some college	58 (20.4)	70 (30.3)	10 (32.3)			70.6 (24.9)	59.7 (25.9)	6.0 (20.9)	
College graduate	58 (20.4)	45 (19.5)	9 (29.0)			59.9 (21.1)	46.4 (20.1)	6.5 (22.4)	
Some graduate school	25 (8.8)	20 (8.7)	0 (0.0)			23.2 (8.2)	18.1 (7.8)	0.0 (0.0)	
Graduate degree	90 (31.7)	62 (26.8)	7 (22.6)			81.3 (28.6)	66.2 (28.7)	12.2 (42.3)	
Race (%)									
White	225 (79.2)	195 (84.4)	20 (64.5)	0.398		230.5 (81.1)	186.4 (80.7)	25.4 (87.8)	0.187
Black	49 (17.3)	31 (13.4)	11 (35.5)			45.8 (16.1)	39.1 (16.9)	3.5 (12.2)	
Other	10 (3.5)	5 (2.2)	0 (0.0)			7.8 (2.7)	5.5 (2.4)	0.0 (0.0)	
History of knee injury (%)									
Left side	86 (30.3)	71 (30.7)	11 (35.5)	0.074		88.7 (31.2)	72.8 (31.5)	7.0 (24.2)	0.110
Right side	97 (34.2)	74 (32.0)	11 (35.5)	0.049		93.8 (33.0)	75.7 (32.8)	7.8 (27.1)	0.087
History of knee surgery (%)									
Left side	44 (15.5)	30 (13.0)	8 (25.8)	0.219		43.2 (15.2)	34.4 (14.9)	3.1 (10.7)	0.091
Right side	44 (15.5)	45 (19.5)	7 (22.6)	0.121		49.7 (17.5)	38.4 (16.6)	4.0 (13.7)	0.069
Physical activity (mean (SD))	167.89 (82.81)	158.98 (79.26)	169.58 (96.21)	0.083		163.52 (82.25)	161.28 (82.07)	136.91 (98.37)	0.197
Family history of OA (%)	25 (8.8)	23 (10.0)	3 (9.7)	0.026		26.7 (9.4)	21.9 (9.5)	3.5 (12.2)	0.060
Radiographic OA (%)	154 ( 54.2)	19 ( 61.3)	125 ( 54.1)	0.097		154.7 (54.5)	12.5 (40.5)	126.4 (54.6)	0.190

Note: IPTW, inverse probability of treatment weighting; SMD, standardized mean difference; BMI, body mass index

Variables selected for matched were selected based on: (1) their potential confounding effects on the association between smoking and OA; and (2) the availability of data in the OAI. Twelve variables were selected and used as IPTW variables in the PS weighting procedure

In the TASOAC cohort, 394 participants (49.7% female, mean age 63.1 ± 7.2 years [range 51.1–79.7]) were enrolled in this study, and the baseline characteristics splitting by smoking status were shown in Additional file 2. Table 2 summarized the baseline characteristics of 366 participants with complete data. The main between-group difference was age, sex, BMI, history of knee injury, and education level. After weighting, all covariates except for education level were balanced (SMD < 0.1).

**Table 2:** Baseline Characteristics of TASOAC cohort, Pre- and Post-IPTW

	*Pre-IPTW*				*Post-IPTW*			
	Never (n = 195)	Former (n = 137)	Current (n = 34)	*SMD*	Never	Former	Current	*SMD*
Age (mean (SD))	62.59 (7.45)	63.93 (6.84)	61.87 (6.51)	0.200	63.02 (7.49)	62.99 (6.81)	62.59 (7.39)	0.040
Sex (%)								
Male	116 (59.5)	57 (41.6)	14 (41.2)	0.248	105.3 (50.9)	75.3 (51.7)	19.8 (49.0)	0.037
Female	79 (40.5)	80 (58.4)	20 (58.8)		101.5 (49.1)	70.3 (48.3)	20.7 (51.0)	
BMI (mean (SD))	27.75 (4.57)	27.65 (4.14)	26.57 (4.02)	0.186	27.65 (4.26)	27.59 (4.27)	27.91 (5.05)	0.047
Education level (%)								
No formal qualifications	24 (12.3)	23 (16.8)	4 (11.8)	0.369	30.1 (14.6)	20.5 (14.1)	5.7 (14.1)	0.210
School or Intermediate certificate	35 (17.9)	33 (24.1)	9 (26.5)		40.5 (19.6)	28.2 (19.4)	9.4 (23.1)	
Higher School or Leaving Certificate	45 (23.1)	22 (16.1)	6 (17.6)		40.9 (19.8)	30.1 (20.7)	8.3 (20.4)	
Trade/apprenticeship	25 (12.8)	20 (14.6)	6 (17.6)		29.1 (14.1)	20.7 (14.2)	4.2 (10.5)	
Certificate/diploma	45 (23.1)	26 (19.0)	8 (23.5)		46.2 (22.4)	32.1 (22.0)	10.5 (25.9)	
University Degree	14 (7.2)	6 (4.4)	1 (2.9)		12.8 (6.2)	9.3 (6.4)	2.5 (6.1)	
Higher University Degree	7 (3.6)	7 (5.1)	0 (0.0)		7.0 (3.4)	4.7 (3.2)	0.0 (0.0)	
Physical activity (mean (SD))	8727.79 (3102.22)	8976.95 (3652.89)	9099.50 (2813.95)	0.079	8767.22 (3208.26)	8819.32 (3394.57)	9071.67 (3142.27)	0.063
History of knee surgery (%)	175 (89.7)	119 (86.9)	29 (85.3)	0.090	182.8 (88.4)	127.9 (87.8)	37.0 (91.2)	0.074
History of knee injury (%)	23 (11.8)	13 (9.5)	2 (5.9)	0.140	22.4 (10.8)	16.8 (11.6)	4.1 (10.0)	0.033
Radiographic OA (%)	118 (60.5)	72 (52.6)	19 (55.9)	0.107	117.3 (56.7)	83.3 (57.2)	23.4 (57.8)	0.014

Note: IPTW: inverse probability of treatment weighting; SMD: standardized mean difference; BMI: body mass index. Variables selected for matched were selected based on: (1) their potential confounding effects on the association between smoking and OA; and (2) the availability of data in the TASOAC. Eight variables were selected and used as IPTW variables in the PS weighting procedure

### Smoking status and OA

In the OAI cohort, smoking status was not associated with the risk of the average rate of tibiofemoral cartilage volume loss over 24 months (Table 3). No statistically significant interactions of smoking status with age in groups and family history of OA were found (all p > 0.1).

**Table 3:** Association between smoking status and cartilage loss of OA

Smoking status	Annual % change in tibiofemoral cartilage volume (n = 546)			Annual % change in tibial cartilage volume (n = 366)		
	*β(%/year)*	95% *CI*	*P*	*β(%/year)*	95% *CI*	*P*
Never	*Reference*	*Reference*	-	*Reference*	*Reference*	-
Former	-0.068	-0.824 to 0.688	0.860	0.001	-0.986 to 0.989	0.998
Current	-0.222	-0.565 to 0.120	0.204	-0.839	-2.520 to 0.844	0.329

Note: CI, confidence interval; ROA, radiographic osteoarthritis. Annual % of change in cartilage volume was calculated using the formula: 100 × [(baseline cartilage volume - follow-up cartilage volume)/baseline cartilage volume]/time between two scans in years

In the TASOAC cohort, smoking status was not associated with the average rate of total tibial cartilage loss (Table 3). No statistically significant interaction between smoking status and age in groups was found (p > 0.1).

Linear regression models for the association between smoking status and cartilage loss were tested and were not violated.

### Sensitivity analyses

The omission of BMI from baseline covariates and multiple imputations for missing data did not change the main results (Additional file 3 & 4).

## Discussion

In this longitudinal study of two community-dwelling cohorts (OAI and TASOAC), we found that smoking was not associated with cartilage volume loss after balancing baseline covariates using an IPTW method. The findings of this study do not support that there is any role of smoking in cartilage loss of knee OA.

The association between smoking and OA is still in debate. Several mechanisms by which smoking may be associated with OA have been proposed. For example, smoking has been shown to impair bone healing [33], and nicotine also diminishes the function of osteoblasts, leading to tissue hypoxia, which induces osteoclast activity and bone resorption. Moreover, an increased systemic inflammation has been found in smokers, which may play an important role in the development of OA [34, 35]. However, inconsistent findings were reported in over 10 prospective cohort studies evaluating the associations of smoking with clinical and radiographic progression of OA [12, 13]. Three studies indicated a protective effect of smoking on the risk of radiographic OA (i.e. KL ≥ 2) [10, 36, 37], and some showed a similar but statistically nonsignificant trend [38–42]. Using data from two longitudinal cohorts, we did not find a statistically significant or clinically important effect of smoking on MRI-detected cartilage volume loss, a more sensitive measure of cartilage loss of OA. The 95% CIs for the association between smoking and annual rate of cartilage loss were mostly within 1%/year, indicating a relatively small effect [43]. While the findings on cartilage loss of OA were consistent, we did not evaluate the association of smoking with symptomatic progression of OA. Despite this, a study from the OAI has shown that smoking was not associated with change in knee symptoms over 72 months [44]. In contrast, some previous studies suggested a protective effect of smoking on risk of clinical OA [40] and total knee replacement (TKR) [41, 42], and some others indicated that smokers were more likely to have painful OA [16] or clinician-diagnosed OA [9]. Thus, the role of smoking in symptomatic progression of OA needs further research.

Obesity is a well-documented risk factor for OA, and smokers often have lower BMI [45, 46]. Therefore, BMI is proposed to be a mediator for the association between smoking and risk of OA, and adjusting for BMI may introduce collider-stratification bias since low BMI may also be induced by other unmeasured factors (e.g., a gene) [14]. In this study, however, the lack of association between smoking and cartilage loss of OA was not changed before and after adjusting for BMI, suggesting a robust result. In addition, no statistically significant interactions of smoking with age group and family history of OA were found. This contrasts to a previous study showing that smoking increased cartilage loss and defects in participants with a family history of OA [17].

The main strength of our study was the inclusion of two independent, community-dwelling cohorts. Moreover, MRI-detected cartilage volume was used to quantify the cartilage loss of OA. Limitations of the current study are worth noting. First, this was an observational study and residual confounding cannot be avoided. However, the IPTW method that we used is superior to the conventional adjustment approach in simulating randomized trials and estimating the causal effect [47]. Besides, some covariates measured in the OAI were not available in the TASOAC, but the consistent findings from the two cohorts suggest that this may not be a significant issue. Second, the intensity of smoke exposure is likely to be dynamic but smoking history was retrospectively obtained and classified into only three categories. Moreover, the duration, frequency, and intensity of smoking or change in smoking status may also play a role in the progression of OA [48]. Third, selection bias may exist in our study, especially in the dataset from the OAI cohort, as indicated by the significant differences between participants selected and not selected from the OAI. Moreover, the between-group imbalances in the OAI cohort were not well controlled even after IPTW. Nonetheless, we have taken these imbalances into account by further adjusting for them. Furthermore, cartilage degeneration is a slow process, and the follow-up duration in this study may not be sufficient to observe significant changes in relation to smoking. Lastly, although we included as many participants as we could in this study, the sample size was modest and formal sample size calculated were not conducted. Therefore, this study may have been underpowered to observe a statistically significant association between smoking and cartilage loss. However, the 95% CI of the results suggested that the association was small and unlikely to be clinically important, as discussed above.

In conclusion, the findings from two independent cohorts consistently showed that smoking was not associated with knee cartilage loss in older adults.

### Electronic supplementary material

Below is the link to the electronic supplementary material.

Additional file 1

Additional file 2

Additional file 3

Additional file 4

Additional file 5

Additional file 6

## Data Availability

The datasets generated and/or analyzed during the current study are available from the corresponding author on reasonable request.

## List of abbreviations

BMI: body mass index
CI: confidence interval
CV: coefficient of variation
IPTW: inverse probability of treatment weighting
JSN: joint space narrowing
MICE: multiple imputation with chained equations
MRI: magnetic resonance imaging
OA: osteoarthritis
OAI: Osteoarthritis Initiative cohort
OARSI: Osteoarthritis Research Society International
PS: propensity scores
SD: standard deviation
SMD: standardized mean difference
STROBE: Strengthening the Reporting of Observational Studies in Epidemiology
TASOAC: Tasmanian Older Adult Cohort
TKR: total knee replacement

## References

[1] Chen L, Yu Y (2020). Exercise and Osteoarthritis. Adv Exp Med Biol.

[2] Disease GBD, Injury I, Prevalence C (2018). Global, regional, and national incidence, prevalence, and years lived with disability for 354 Diseases and injuries for 195 countries and territories, 1990–2017: a systematic analysis for the global burden of Disease Study 2017. Lancet.

[3] Sun X, Zhen X, Hu X, Li Y, Gu S, Gu Y et al. Osteoarthritis in the Middle-aged and Elderly in China: prevalence and influencing factors. Int J Environ Res Public Health. 2019;16(23). 10.3390/ijerph16234701 PMC6926632 31779104

[4] Hulshof CTJ, Pega F, Neupane S, van der Molen HF, Colosio C, Daams JG (2021). The prevalence of occupational exposure to ergonomic risk factors: a systematic review and meta-analysis from the WHO/ILO Joint Estimates of the work-related Burden of Disease and Injury. Environ Int.

[5] Boer CG, Hatzikotoulas K, Southam L, Stefansdottir L, Zhang Y, Coutinho de Almeida R (2021). Deciphering osteoarthritis genetics across 826,690 individuals from 9 populations. Cell.

[6] Gessl I, Popescu M, Schimpl V, Supp G, Deimel T, Durechova M (2021). Role of joint damage, malalignment and inflammation in articular tenderness in rheumatoid arthritis, psoriatic arthritis and osteoarthritis. Ann Rheum Dis.

[7] Malhotra J, Malvezzi M, Negri E, La Vecchia C, Boffetta P (2016). Risk factors for Lung cancer worldwide. Eur Respir J.

[8] Münzel T, Hahad O, Kuntic M, Keaney JF, Deanfield JE, Daiber A (2020). Effects of Tobacco cigarettes, e-cigarettes, and waterpipe Smoking on endothelial function and clinical outcomes. Eur Heart J.

[9] Ni J, Wang P, Yin KJ, Huang JX, Tian T, Cen H (2022). Does Smoking protect against developing osteoarthritis? Evidence from a genetically informed perspective. Semin Arthritis Rheum.

[10] Felson DT, Anderson JJ, Naimark A, Hannan MT, Kannel WB, Meenan RF (1989). Does Smoking protect against osteoarthritis?. Arthritis Rheum.

[11] Anderson JJ, Felson DT (1988). Factors associated with osteoarthritis of the knee in the first national Health and Nutrition Examination Survey (HANES I). Evidence for an association with overweight, race, and physical demands of work. Am J Epidemiol.

[12] Hui M, Doherty M, Zhang W (2011). Does Smoking protect against osteoarthritis? Meta-analysis of observational studies. Ann Rheum Dis.

[13] Kong L, Wang L, Meng F, Cao J, Shen Y (2017). Association between Smoking and risk of knee osteoarthritis: a systematic review and meta-analysis. Osteoarthr Cartil.

[14] Felson DT, Zhang Y (2015). Smoking and osteoarthritis: a review of the evidence and its implications. Osteoarthritis Cartilage.

[15] Guermazi A, Roemer FW, Burstein D, Hayashi D (2011). Why radiography should no longer be considered a surrogate outcome measure for longitudinal assessment of cartilage in knee osteoarthritis. Arthritis Res Ther.

[16] Amin S, Niu J, Guermazi A, Grigoryan M, Hunter DJ, Clancy M (2007). Cigarette Smoking and the risk for cartilage loss and knee pain in men with knee osteoarthritis. Ann Rheum Dis.

[17] Ding C, Cicuttini F, Blizzard L, Jones G (2007). Smoking interacts with family history with regard to change in knee cartilage volume and cartilage defect development. Arthritis Rheum.

[18] von Elm E, Altman DG, Egger M, Pocock SJ, Gøtzsche PC, Vandenbroucke JP (2007). The strengthening the reporting of Observational studies in Epidemiology (STROBE) statement: guidelines for reporting observational studies. Lancet.

[19] Zhai G, Blizzard L, Srikanth V, Ding C, Cooley H, Cicuttini F (2006). Correlates of knee pain in older adults: tasmanian older adult cohort study. Arthritis Rheum.

[20] Dam EB, Lillholm M, Marques J, Nielsen M (2015). Automatic segmentation of high- and low-field knee MRIs using knee image quantification with data from the osteoarthritis initiative. J Med Imaging (Bellingham Wash).

[21] Cai G, Jiang M, Cicuttini F, Jones G (2019). Association of age, sex and BMI with the rate of change in tibial cartilage volume: a 10.7-year longitudinal cohort study. Arthritis Res Therapy.

[22] O’Brien MS, McDougall JJ (2019). Age and frailty as risk factors for the development of osteoarthritis. Mech Ageing Dev.

[23] Schlenk EA, Fitzgerald GK, Rogers JC, Kwoh CK, Sereika SM (2021). Promoting physical activity in older adults with knee osteoarthritis and Hypertension: a Randomized Controlled Trial. J Aging Phys Act.

[24] Siviero P, Limongi F, Gesmundo A, Zambon S, Cooper C, Dennison EM (2021). Factors Associated with Functional decline in Hand and Hip/Knee Osteoarthritis after one year: data from a Population-based study. Arthritis Care Res (Hoboken).

[25] Kellgren JH, Lawrence JS (1957). Radiological assessment of osteo-arthrosis. Ann Rheum Dis.

[26] Washburn RA, Smith KW, Jette AM, Janney CA (1993). The physical activity scale for the Elderly (PASE): development and evaluation. J Clin Epidemiol.

[27] Dore DA, Winzenberg TM, Ding C, Otahal P, Pelletier JP, Martel-Pelletier J (2013). The association between objectively measured physical activity and knee structural change using MRI. Ann Rheum Dis.

[28] Altman RD, Hochberg M, Murphy WA, Wolfe F, Lequesne M (1995). Atlas of individual radiographic features in osteoarthritis. Osteoarthritis Cartilage.

[29] Stuart EA (2010). Matching methods for causal inference: a review and a look forward. Stat Science: Rev J Inst Math Stat.

[30] Williamson EJ, Forbes A, White IR (2014). Variance reduction in randomised trials by inverse probability weighting using the propensity score. Stat Med.

[31] van der Wal WM, Geskus RB (2011). Ipw: an R package for inverse probability weighting. J Stat Softw.

[32] Cole SR, Hernán MA (2008). Constructing inverse probability weights for marginal structural models. Am J Epidemiol.

[33] Hollinger JO, Schmitt JM, Hwang K, Soleymani P, Buck D (1999). Impact of nicotine on bone healing. J Biomedical Mater Research: Official J Soc Biomaterials Japanese Soc Biomaterials Australian Soc Biomaterials.

[34] Bazzano LA, He J, Muntner P, Vupputuri S, Whelton PK (2003). Relationship between cigarette Smoking and novel risk factors for Cardiovascular Disease in the United States. Ann Intern Med.

[35] Bermudez EA, Rifai N, Buring JE, Manson JE, Ridker PM (2002). Relation between markers of systemic vascular inflammation and Smoking in women. Am J Cardiol.

[36] Felson DT, Zhang Y, Hannan MT, Naimark A, Weissman B, Aliabadi P (1997). Risk factors for incident radiographic knee osteoarthritis in the elderly: the Framingham Study. Arthritis Rheum.

[37] Szoeke CEI, Cicuttini FM, Guthrie JR, Clark MS, Dennerstein L (2006). Factors affecting the prevalence of osteoarthritis in healthy middle-aged women: data from the longitudinal Melbourne Women’s midlife Health Project. Bone.

[38] Hootman JM, Macera CA, Helmick CG, Blair SN (2003). Influence of physical activity-related joint stress on the risk of self-reported hip/knee osteoarthritis: a new method to quantify physical activity. Prev Med.

[39] Engström G, Gerhardsson de Verdier M, Rollof J, Nilsson PM, Lohmander LS (2009). C-reactive protein, metabolic syndrome and incidence of severe hip and knee osteoarthritis. A population-based cohort study. Osteoarthr Cartil.

[40] Toivanen AT, Heliovaara M, Impivaara O, Arokoski JP, Knekt P, Lauren H (2010). Obesity, physically demanding work and traumatic knee injury are major risk factors for knee osteoarthritis–a population-based study with a follow-up of 22 years. Rheumatology (Oxford).

[41] Mnatzaganian G, Ryan P, Reid CM, Davidson DC, Hiller JE (2013). Smoking and primary total hip or knee replacement due to osteoarthritis in 54,288 elderly men and women. BMC Musculoskelet Disord.

[42] Leung YY, Ang LW, Thumboo J, Wang R, Yuan JM, Koh WP (2014). Cigarette Smoking and risk of total knee replacement for severe osteoarthritis among Chinese in Singapore–the Singapore Chinese health study. Osteoarthr Cartil.

[43] Cicuttini FM, Jones G, Forbes A, Wluka AE (2004). Rate of cartilage loss at two years predicts subsequent total knee arthroplasty: a prospective study. Ann Rheum Dis.

[44] Dube CE, Liu SH, Driban JB, McAlindon TE, Eaton CB, Lapane KL (2016). The relationship between Smoking and knee osteoarthritis in the Osteoarthritis Initiative. Osteoarthr Cartil.

[45] Wluka AE, Lombard CB, Cicuttini FM (2013). Tackling obesity in knee osteoarthritis. Nat Rev Rheumatol.

[46] Albanes D, Jones DY, Micozzi MS, Mattson ME (1987). Associations between Smoking and body weight in the US population: analysis of NHANES II. Am J Public Health.

[47] Austin PC, Stuart EA (2015). Moving towards best practice when using inverse probability of treatment weighting (IPTW) using the propensity score to estimate causal treatment effects in observational studies. Stat Med.

[48] Mnatzaganian G, Ryan P, Norman PE, Davidson DC, Hiller JE (2011). Smoking, body weight, physical exercise, and risk of lower limb total joint replacement in a population-based cohort of men. Arthritis Rheum.

